# Robust Microarray Meta-Analysis Identifies Differentially Expressed Genes for Clinical Prediction

**DOI:** 10.1100/2012/989637

**Published:** 2012-12-18

**Authors:** John H. Phan, Andrew N. Young, May D. Wang

**Affiliations:** ^1^Department of Biomedical Engineering, Georgia Institute of Technology and Emory University, 313 Ferst Drive, Atlanta, GA 30332, USA; ^2^Department of Pathology and Laboratory Medicine, Emory University School of Medicine, Grady Health System, Grady Memorial Hospital, Atlanta, GA 30303, USA

## Abstract

Combining multiple microarray datasets increases sample size and leads to improved reproducibility in identification of informative genes and subsequent clinical prediction. Although microarrays have increased the rate of genomic data collection, sample size is still a major issue when identifying informative genetic biomarkers. Because of this, feature selection methods often suffer from false discoveries, resulting in poorly performing predictive models. We develop a simple meta-analysis-based feature selection method that captures the knowledge in each individual dataset and combines the results using a simple rank average. In a comprehensive study that measures robustness in terms of clinical application (i.e., breast, renal, and pancreatic cancer), microarray platform heterogeneity, and classifier (i.e., logistic regression, diagonal LDA, and linear SVM), we compare the rank average meta-analysis method to five other meta-analysis methods. Results indicate that rank average meta-analysis consistently performs well compared to five other meta-analysis methods.

## 1. Introduction

We develop a simple, yet robust meta-analysis-based feature selection (FS) method for microarrays that ranks genes by differential expression within several independent datasets,then combines the ranks using a simple average to produce a final list of rank-ordered genes. Such meta-analysis methods can increase the power of microarray data analysis by increasing sample size [1]. The subsequent improvement to differentially expressed gene (DEG) detection, or to FS is essential for downstream clinical applications. Many of these applications, such as disease diagnosis and disease subtyping, are predictive in nature and are important for guiding therapy. However, DEG detection can be difficult due to technical and biological noise or due to small sample sizes relative to large feature sizes [2]. These properties are typical of many microarray datasets. Despite small sample sizes, the number of gene expression datasets available to the research community has grown [3]. Thus, it is important to develop methods that can use all available knowledge by simultaneously analyzing several microarray datasets of similar clinical focus. However, combining high-throughput gene expression datasets can be difficult due to technological variability. Differences in microarray platform [4] or normalization and preprocessing methods [5] affect the comparability of gene expression values. Laboratory batch effects can also affect reproducibility [6]. Numerous studies have proposed novel strategies to remove batch effects [7]. However, in some cases, batch effect correction can have undesirable consequences [8]. In light of these challenges, several studies have proposed novel methods for meta-analysis of multiple microarray datasets.

Existing microarray meta-analysis methods either combine separate statistics for each gene expression dataset or aggregate samples into a single large dataset to estimate global gene expression. The study by Park et al. used analysis of variance to identify unwanted effects (e.g., the effect of different laboratories) and modeled these effects to detect DEGs [9]. Choi et al. used a similar approach to compute an “effect size” quantity, representing a measure of precision for each study, and used this “effect size” to directly compare and combine microarray datasets [10]. Wang et al. combined the fold change of genes between classes from three microarray datasets and weighted each dataset by its variance such that datasets with higher variance contribute less to the final statistic [11]. Yoon et al. conducted a large-scale study of gene expression by examining the variation of genes across multiple microarray datasets, regardless of the clinical focus [12]. Breitling and Herzyk ranked fold changes between all interclass pairs of samples and computed the product of all ranks for each gene [13]. More recently, Campain and Yang reviewed several meta-analysis methods and assessed their performance using both classification accuracy and synthetic data [14]. Research has shifted towards methods that consider multiple FS methods, reflecting the fact that no single FS method performs well for all datasets [15]. Although several meta-analysis methods exist, except for the study by Campain and Yang, the literature rarely compares these methods in a comprehensive manner.

**Figure 1:**
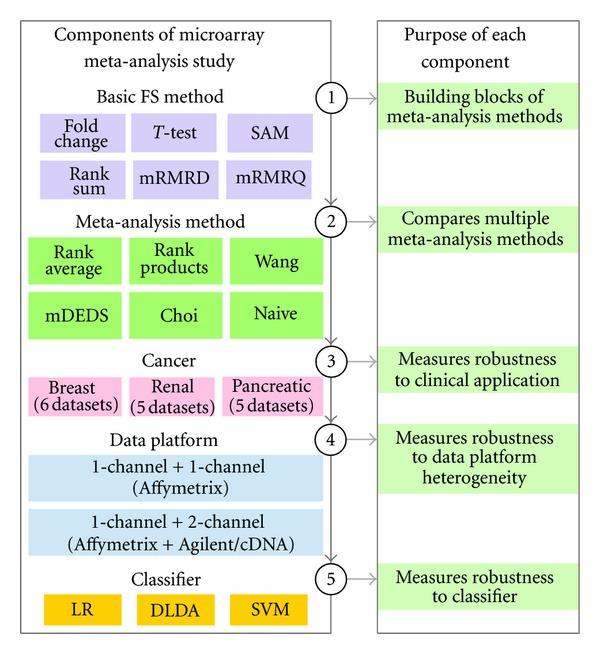
Study design diagram. We compare the predictive performance of meta-analysis-based feature selection (FS) methods by designing a study that considers five components: (1) basic FS methods that are the building blocks of some of the meta-analysis methods, (2) meta-analysis-based FS methods, (3) clinical application, (4) microarray data platform, and (5) classifier (logistic regression, diagonal LDA and linear SVM). Since the “best” meta-analysis-based FS method may be dataset- or application-specific, assessing performance over a wide variety of factors enables an evaluation of the method's robustness.

We develop the rank average method, a simple meta-analysis-based FS method, for identifying DEGs from multiple microarray datasets and design a study (Figure 1) to compare rank average to five other meta-analysis-based FS methods. We focus on the predictive ability of genes emerging from meta-analysis and show that rank average meta-analysis is robust with respect to three factors. These three factors are (1) clinical application (i.e., breast, renal, and pancreatic cancer diagnosis or subtyping), (2) data platform heterogeneity (i.e., combining different microarray platforms), and (3) classifier. Using a comprehensive factorial analysis, we rate each meta-analysis-based FS method relative to its peers. In terms of identifying genetic features with reproducible predictive performance and in terms of robustness to multiple factors, results indicate that rank average meta-analysis performs consistently well in comparison to five other meta-analysis-based FS methods.

## 2. Methods

### 2.1. Microarray Datasets

We use six breast cancer, five renal cancer, and five pancreatic cancer gene expression datasets (Table 1) to compare meta-analysis-based FS methods. Each renal cancer dataset examines patient samples from several subtypes of tumors: clear cell (CC), oncocytoma (ONC), chromophobe (CHR), and papillary (PAP). We are interested in identifying genes differentially expressed between the CC subtype and all other subtypes, that is, CC versus ONC/CHR/PAP. These renal cancer datasets share a similar clinical focus. However, they are heterogeneous in terms of microarray platform [16–21]. Similarly, the breast cancer datasets are heterogeneous in both platform and clinical focus [22–26]. Although patient samples from each dataset have undergone different treatment for breast cancer and have been extracted at different stages of the disease, each sample is labeled as either estrogen receptor positive (ER+) or negative (ER−). Thus, we assess the performance of classifiers that predict the estrogen receptor status. The pancreatic cancer datasets also include a variety of platforms and clinical focuses [27–31]. We identify genes to discriminate pancreatic cancer versus noncancer patient samples. These datasets contain different numbers of probes (or probesets in the case of Affymetrix datasets) due to differences in microarray platform. Within each dataset group, we reduce the number of probes in each dataset to a common shared set based on probe sequence similarity.

### 2.2. Rank Average Meta-Analysis

The meta-analysis-based FS method proposed in this paper ranks genes individually in each dataset and computes the average rank of each gene. Gene rank order is determined by a measure of differential expression (which can be any of a number of basic FS methods such as fold change or *t*-test) and we assume that this rank order is invariant to batch effects. Using the average rank of a gene across several datasets to obtain the final multidataset rank order, we can infer (1) the relative strength of that gene in differentiating the patient samples of interest and (2) the consistency of the gene's differential expression across multiple studies.

The remainder of this section uses the following mathematical notation. *K* is the total number of datasets, *M* is the total number of genes in each dataset, and *N*_*k*_ is the number of samples in dataset *k*, where *k* = 1 ⋯ *K* and *N* is the total number of samples in all datasets. We denote a gene *i* in dataset *k* as a vector

(1) $$\begin{array}{c} {\overset{-}{g}}_{i,k}=\left(x_{1}^{i,k},x_{2}^{i,k},\ldots ,x_{N_{k}}^{i,k}\right), \end{array}$$

where *x*_*j*_^*i*,*k*^ is the expression value of gene *i* of sample *j* in dataset *k*. In the case of sample aggregation (i.e., the naive method of meta-analysis), we denote a gene *i* across all datasets with

(2) g−i,•=((x1i,1,x2i,1,…,xN1i,1),(x1i,2,x2i,2,…,xN2i,2),…, (x1i,K,x2i,K,…,xNKi,K)).

Using this notation, we can define a function, $r_{i,k,\vartheta }=R_{\vartheta }({\overset{-}{g}}_{i,k})$, to compute the rank, *r*_*i*,*k*,*ϑ*_, of a gene, ${\overset{-}{g}}_{i,k}$, using a ranking algorithm denoted by *ϑ*. A smaller rank indicates a greater degree of differential expression. In the case of sample aggregation, the ranking function takes the form $r_{i,•,\vartheta }=R_{\vartheta }({\overset{-}{g}}_{i,•})$. The average rank, ${\overset{-}{r}}_{i}$, of a gene *i* across all datasets, weighted by number of samples in each dataset, *N*_*k*_, is

(3) $$\begin{array}{c} {\overset{-}{r}}_{i}=\frac{1}{N}\sum_{k=1}^{K}N_{k}R_{\vartheta_{k}}\left({\overset{-}{g}}_{i,k}\right). \end{array}$$

Weighting gives preference to ranks from datasets with larger sample sizes.

We consider several basic FS, or gene ranking, methods as follows: fold change (FC), *t*-test (*T*), significance analysis of microarrays (SAM) [32], rank-sum (RS), minimum redundancy maximum relevance using the difference formulation (mRMRD), and mRMR using the quotient formulation (mRMRQ) [33]. We explicitly define the rank algorithm for the *k*th dataset as

(4) $$\begin{array}{c} \vartheta_{k}\in \left\{\text{FC},T,\text{SAM},\text{RS},\text{mRMRD},\text{mRMRQ}\right\}. \end{array}$$

For each dataset and each basic FS method, we use three-fold cross-validation to compute an estimate of classification performance (measured using AUC) averaged over 20 feature sizes (ranging from the top single feature to the top twenty features). We then choose the basic FS method, *ϑ*_*k*_, with highest estimated classification performance for each dataset. Because each basic FS method makes different assumptions about DEGs and the correctness of these assumptions varies from dataset to dataset, allowing a different basic FS method for each dataset can improve performance.

### 2.3. Predictive Performance

**Figure 2:**
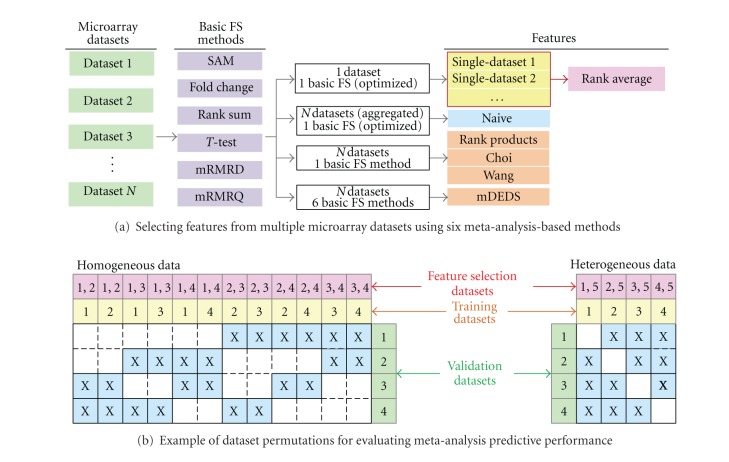
Procedure for comparing the predictive performance of six microarray meta-analysis-based FS methods. (a) Features are selected from microarray datasets using the rank average meta-analysis method (pink box), several other meta-analysis methods (orange boxes: mDEDS, rank products, Choi, and Wang), and a naive method (blue box) that aggregates samples into a larger dataset. Rank average meta-analysis chooses a single feature selection (FS) method from among several basic FS methods (SAM, fold change, rank sum, *t*-test, mRMRD, and mRMRQ) for each individual dataset that optimizes prediction performance (via cross-validation) over the top 20 features. A simple weighted average of gene ranks from all individual datasets produces the final set of rank average meta-analysis features. The rank products, Choi, and Wang methods use one basic FS method to select features from multiple datasets while the mDEDS method uses all six basic FS methods. (b) Features are selected from two or more datasets from each group to build a classifier (pink boxes), which is trained with samples from only one dataset (yellow boxes). The performance of the classifier is assessed using independent datasets (datasets not used for training or feature selection, green boxes). The predictive performance of a microarray meta-analysis-based FS method is an average over all permutations of training and validation datasets (blue boxes). In the example, datasets 1–4 consist of one-channel Affymetrix arrays while dataset 5 (in the case of heterogeneous data) consists of two-channel arrays.

We use classification performance to assess meta-analysis-based FS methods with the assumption that improved FS leads to higher prediction performance when classifying samples from an independent dataset. We assess prediction performance using independent training and testing datasets because of the small sample size of some of the datasets and because we want to reflect clinical scenarios in which predictive models would likely be derived from data collected from a separate batch of patients. We compare our proposed rank average meta-analysis method to other meta-analysis methods including: (1) the rank products method [13], (2) the mDEDS method [14], (3) Choi et al.'s method of interstudy variability [10], (4) Wang et al.'s method of weighting differential expression by variance [11], and (5) a naive method that aggregates samples from multiple datasets. The rank products, mDEDS, Choi, and Wang methods can be applied to multiple datasets as well as to single datasets. For each method and each dataset group, we compute single-dataset performance, combined homogeneous-dataset performance (from two to four datasets combined), and combined heterogeneous-dataset performance (Figure 2(a)).

Classification performance depends on both feature selection and number of samples available for training. We are interested in performance gains due to meta-analysis-based FS alone. We isolate this performance gain by training classifiers with samples from a single dataset only, while allowing the features used for training to come from multiple datasets. Thus, any improvement (or degradation) in classification performance of a meta-analysis-based FS method in comparison to the baseline single-dataset FS is due to features selected rather than to increases in training sample size. We assess classification performance using a separate validation dataset and permute the datasets such that each individual dataset in each dataset group—renal, breast, and pancreatic cancer—is used at least once for validation. Moreover, for each permutation, we use 100 iterations of bootstrap sampling from the training datasets to estimate classification performance.  Figure 2(b) is an example of the permutations possible with a five-dataset group (datasets 1–4 are the same platform while dataset 5 is a different platform), in which the prediction performance of two-dataset combination is assessed. This procedure can be expanded to handle three-dataset, four-dataset, or higher combinations for FS.

The procedure for measuring predictive performance of heterogeneous-dataset combination is slightly different. Each dataset group contains several one-channel Affymetrix datasets and one two-channel dataset (either cDNA or Agilent). Gene expression values of the two-channel datasets are computed as log ratios, resulting in different dynamic ranges compared to the one-channel datasets. We assess the robustness of each meta-analysis-based FS method to heterogeneous data platforms by first determining the performance of the method when combining only Affymetrix data (Figure 2(b), homogeneous data), then comparing to results obtained when combining a mixture of Affymetrix and two-channel arrays (Figure 2(b), Heterogeneous Data). For example, we compute heterogeneous combination performance by combining one or more Affymetrix datasets to the two-channel dataset, then training a classifier using one of the Affymetrix datasets, and testing samples from an independent dataset (again Affymetrix). Thus, not only should a good meta-analysis-based FS method perform well with respect to single dataset FS, but also the method should exhibit minimal performance degradation, if any, when combining heterogeneous data platforms.

## 3. Results

### 3.1. Robustness of Rank Average Meta-Analysis

**Figure 3:**
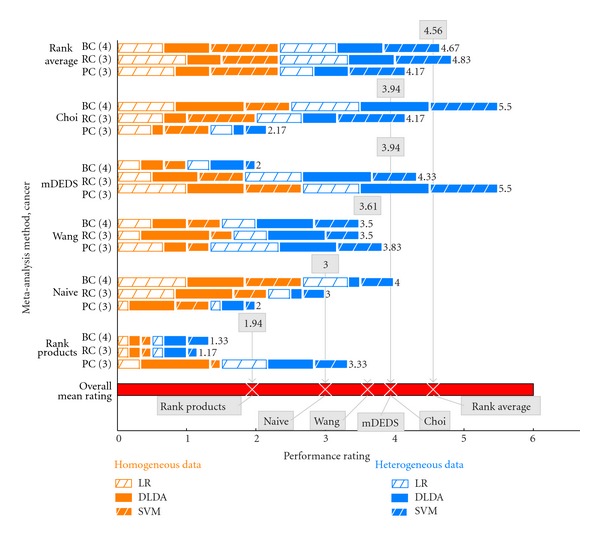
Rating meta-analysis methods by prediction performance when combining all available datasets. Each meta-analysis method (rank average, rank products, Wang, mDEDS, Choi, and naive) is rated relative to its peers. We assess performance rating across three factors: (1) clinical application (breast cancer: BC, renal cancer: RC, and pancreatic cancer: PC), (2) data platform heterogeneity (homogeneous: orange, heterogeneous: blue), and (3) classifier (logistic regression: LR, diagonal LDA: DLDA and linear SVM). For each combination of factors, the rating of each meta-analysis method is represented by an additive bar. Methods with higher absolute prediction performance receive higher ratings (and longer bars). When considering absolute prediction performance, rank average, with a mean overall rating of 4.56, performs consistently well compared to its peers.

We rate each meta-analysis method by absolute prediction performance (Figure 3). Based on this criterion, we find that rank average meta-analysis, with the highest overall mean rating of 4.56, performs consistently well compared to five other meta-analysis methods including the mDEDS, rank products, Choi, Wang, and naive methods. This analysis answers the question: which meta-analysis-based FS method consistently exhibits the largest prediction performance when combining all available datasets? We assign a rating to each meta-analysis method for every combination of three factors that include (1) clinical application or dataset group, (2) data platform heterogeneity (combining similar or different microarray platforms), and (3) classifiers (logistic regression: LR, diagonal linear discriminant: DLDA, and linear SVM). Ratings for each meta-analysis method are relative to its peers, with higher ratings indicating better prediction performance under the same combination of factors. In Figure 3, bars are proportional to performance ratings. Using pancreatic cancer (PC) as an example, the rank average meta-analysis method has a rating of five (corresponding to a predictive performance AUC of 81.5, See Supplemental Table S1 available online at doi:10.1100/2012/989637) when analyzing homogeneous datasets and when using the logistic regression classifier. This means that its absolute prediction performance is higher than that of four other meta-analysis methods compared under the same conditions (i.e., homogeneous data, logistic regression classifier). The results illustrated in Figure 3 and obtained through a comprehensive analysis of three factors suggest that, relative to its peers, rank average meta-analysis is robust when considering absolute prediction performance.

### 3.2. Rank Average Identifies Biologically Sensible Genes

For each dataset group, we combine all available microarray datasets and use the rank average meta-analysis method to identify DEGs. Assessing DEG detection performance by examining the genes is difficult unless we know, via validation, whether or not these genes are truly differentially expressed. However, because of the sheer number of genes in high-throughput datasets, the validation process is often time and resource intensive. Despite this, we examine the top ranked genes from each dataset group to verify that the rank average meta-analysis method is identifying genes that are biologically sensible.

Table 2 lists the top 20 genes selected from meta-analysis of each of the three dataset groups: six breast cancer, five renal cancer, and five pancreatic cancer datasets. We optimize the FS method for each individual dataset using three-fold cross validation and the diagonal LDA classifier. The optimal FS method for each dataset differs. We compare ER+ and ER− samples for each breast cancer dataset and find, not surprisingly, that the ESR1 gene (estrogen receptor) is the top ranked gene for all but one dataset. Accordingly, the weighted average rank of ESR1 places it at the top of the combined list. Among the other genes in the list, NAT1 [34], DNALI1, SCUBE2 [35], and TFF1 [36] have been implicated in breast cancer. Although the individual dataset ranks of these genes vary from low to relatively high ranks (e.g., 200 to 300), it is the consistency of selecting these genes from multiple datasets that places them at the top of the combined list. In Table 2, we include the number of individual datasets in which the gene is ranked in the top 20. 

We compare the renal cancer clear cell subtype to three other subtypes (i.e., chromophobe, oncocytoma, and papillary) to identify DEGs. The top gene we identify is LOX, which is an oncogene implicated in clear cell renal cancer [37]. The ADFP gene, ranked at #3 in the combined list, is especially interesting because it may be a potential urinary biomarker for detecting renal cancer [38]. ADFP is ranked favorably in all but the Higgins dataset, in which it is ranked at number 75.

The rank average meta-analysis method identifies S100P as the top pancreatic cancer gene, which has been implicated in several studies [39, 40]. The S100P gene has a relatively favorable ranking in the Pilarsky and Pei datasets and moderate to un-favorable rankings in the other datasets, indicating that analysis of individual datasets may not readily identify the gene. Another example, LAMC2, is ranked favorably in the Ishikawa and Pei datasets, but relatively higher in the other datasets. Overall, LAMC2 is ranked second in the combined results and is, according the to literature, a purported pancreatic cancer gene [41]. Weighted average ranks for the pancreatic cancer results increase quickly compared to the breast and renal cancer results, indicating increased heterogeneity among the ranks of the individual datasets. One explanation for this is the slight difference in dataset subtype comparisons. For example, one of the datasets, Ishikawa, extracted RNA samples from pancreatic juice rather than from solid tumors.

The degree of differential expression (and consequently, the rank) of a gene can vary significantly from dataset to dataset. Combining DEG detection results by averaging ranks across datasets reduces variability and improves statistical confidence. Analysis of a single microarray dataset may result in errors during DEG detection—for example, false positives and false negatives (genes that should be differentially expressed, but not favorably ranked). In general, these errors can be reduced by increasing sample size. Combining microarray datasets by averaging ranks effectively increases sample size while enabling robust analysis of heterogeneous data.

## 4. Discussion

In order to understand the differences in performance among the six meta-analysis-based FS methods, we identify and list the differences and similarities in Table 3. We focus on three properties: (a) basic FS methods forming the basis of meta-analysis, (b) the manner in which these basic FS methods are chosen and applied to individual microarray datasets, and (c) the use of ranks.

Among the five meta-analysis methods (not including the naive control method) rank average and mDEDS are the only methods that consider multiple basic FS methods—for example, fold change, *t*-statistic, SAM, and rank sum—for detecting DEGs (Table 3, row 1). The rank products, Choi and Wang methods use modified forms of basic FS methods. Moreover, rank average is the only method that chooses one basic FS method for each dataset to maximize prediction performance (Table 3, row 2). In contrast, mDEDS uses all of the available basic FS methods for each dataset. Finally, rank average and rank products are the only meta-analysis methods that are rank-based (Table 3, row 3).

Among the basic FS methods, no method can be considered the best because of the data-dependent nature of microarray analysis. Thus, rank average and mDEDS benefit by considering multiple basic FS methods. However, some basic FS methods can produce erroneous results when inappropriately applied (e.g., using a *t*-statistic with gene expression data that is not normally distributed). Rank average meta-analysis further benefits from selecting a single basic FS that optimizes prediction performance. On the other hand, the performance of mDEDS meta-analysis can degrade if it includes a basic FS method that is incompatible with the data. Likewise, the performance of rank products can degrade when the fold change FS method is not appropriate for the data. The Choi and Wang methods may also suffer from this problem. However, they seem to perform fairly well when applied to the datasets in this study (see Figure 3). Finally, rank-based meta-analysis methods that consider multiple basic FS methods allow a fair comparison among the basic FS methods. In light of these results, for microarray meta-analysis, we recommend (1) to use rank-based methods, (2) to consider a wide variety of basic FS methods, and (3) to optimize the FS method for each individual dataset based on application-specific criteria (e.g., prediction performance for diagnostic applications).

Despite the benefits summarized in Table 3, rank average meta-analysis and the evaluation criteria presented in this study are not without limitations. The limitations of this study include (1) the scope of data and classifiers considered, (2) the criterion for measuring performance of a meta-analysis method, and (3) normalization and pre-processing of gene expression data. First, the results of this study may be dataset-specific. Although we have strived to provide a wide range of scenarios to allow adequate assessment of these meta-analysis methods, results may differ when applied to other dataset groups. Second, we use prediction AUC as the performance criterion. However, microarray-based clinical prediction is only one possible application. Other applications may need to identify genes based on biological relevance [15]. It is unclear which meta-analysis methods would perform well in such applications. The rank average meta-analysis method benefits from choosing a basic FS method for each individual dataset that optimizes (via cross-validation) prediction performance. Thus, there is a potential bias in the performance of rank average meta-analysis. On the other hand, the ability to choose basic FS methods that perform well for a particular application, such as prediction, could be considered a favorable property of rank average meta-analysis. Finally, it is possible that normalization of gene expression datasets (e.g., quantile normalization) can improve the performance of meta-analysis by reducing batch effect. Specifically, removal of batch effects (1) can improve prediction performance when training and testing are applied to independent, heterogeneous datasets and (2) can improve the performance of simple meta-analysis methods that aggregate samples from multiple heterogeneous datasets. However, we do not consider any batch-effect normalization procedures in this study. 

## 5. Conclusions

In order to address the sample-size problem in gene expression analysis as well as the need for accurate solutions for clinical prediction problems, we proposed the rank average meta-analysis-based FS method. Rank average meta-analysis identifies differentially expressed genes from multiple microarray datasets. We used a comprehensive study of multiple factors and found that rank average performs consistently well compared to five other meta-analysis methods in terms of prediction performance. This comprehensive study enabled us to measure the robustness of rank average to three factors that are often encountered in clinical prediction applications. These factors include clinical application (e.g., breast, renal, and pancreatic cancer), microarray data platform heterogeneity, and classifier model (logistic regression, diagonal LDA, and SVM). Rank average meta-analysis, performs well because it selects dataset-specific basic FS methods and then averages the ranks across all individual datasets to produce a final robust gene ranking. In comparison to five other meta-analysis methods the rank average method is not always the best method for some factor combinations. However, it is consistently among the best performing in terms of its ability to identify predictive genes. Although we presented results from analysis of microarray gene expression data, the proposed methods may be generalized for other bioinformatics problems that require feature selection.

## Supplementary Material

Supplemental Table S1 – Rating meta-analysis methods by prediction performance when combining all available datasets. This table lists the predictive performance (AUC, area under the ROC curve x 100) for each clinical application (breast cancer, renal cancer, and pancreatic cancer), data platform heterogeneity (Hom: Homogeneous, Het: Heterogeneous), and classifier (LR: Logistic Regression, DLDA: Diagonal LDA, and Linear SVM). The numbers in parentheses indicate the performance rating relative to other meta-analysis methods (rated horizontally, higher is better). A mean rating is computed for each clinical application and each meta-analysis method across all combinations of data platform heterogeneity and classifier. An overall mean rating is computed for each meta-analysis method. Ratings are proportional to bar lengths in Figure 3.

## Figures and Tables

**Table:** (a) Breast cancer estrogen receptor status

Dataset	ER+	ER−	Platform	No. of probes
MDACC Train	80	50	Affy HG-U133A	22283
MDACC Test	60	40	Affy HG-U133A	22283
Miller	213	34	Affy HG-U133A	22283
Sotiriou	72	24	Affy HG-U133A	22283
Minn	57	42	Affy HG-U133A	22283
Van't Veer	226	69	Agilent 2-Color	24496

Common probes: 8953.

**Table:** (b) Renal cancer subtype

Dataset	CC	Other	Platform	No. of probes
Schuetz	13	12	Affy HG-Focus	8793
Jones	32	29	Affy HG-U133A	22283
Kort	10	30	Affy HG-U133+2.0	54675
Yusenko	26	27	Affy HG-U133+2.0	54675
Higgins	26	9	cDNA 2-Color	22689

Common probes: 946.

**Table:** (c) Pancreatic cancer diagnosis

Dataset	Normal	Cancer	Platform	No. of probes
Badea	39	39	Affy HG-U133+2.0	54675
Ishikawa	25	24	Affy HG-U133A/B	44928
Pei	16	36	Affy HG-U133+2.0	54675
Pilarsky	18	27	Affy HG-U133A/B	44928
Iacobuzio-Donahue	5	17	cDNA 2-Color	43910

Common probes: 4530.

**Table 2:** Differentially expressed genes identified from rank average meta-analysis of multiple microarray datasets.

Breast cancer			Renal cancer			Pancreatic cancer		
Gene symbol	Weighted average rank	Top 20 in # of datasets	Gene symbol	Weighted average rank	Top 20 in # of datasets	Gene symbol	Weighted average rank	Top 20 in # of datasets
ESR1	0.20	6	LOX	13.65	4	S100P	31.42	2
NAT1	33.99	3	COL5A2	16.86	3	LAMC2	51.44	2
DNALI1	48.46	1	ADFP	19.08	4	PHLDA2	201.93	1
SCUBE2	69.27	1	SCNN1A	19.25	2	S100A2	233.07	0
TFF1	76.74	1	LOXL2	21.37	3	MSLN	234.39	1
MYB	82.17	0	ELTD1	27.17	4	WFDC2	236.00	1
CYP2B7P1	86.93	1	PPARGC1A	30.73	1	ITGB6	238.13	0
PDZK1	98.81	0	IFITM1	31.19	2	HK2	239.87	2
PADI2	114.44	0	RALGPS1	37.17	2	R88990*	244.34	0
DNAJC12	123.83	0	VWF	37.85	2	ANO1	252.57	1
TSPAN1	126.87	0	CD70	41.65	0	MXRA5	261.28	0
CDH3	127.46	1	ARHGDIB	42.60	1	PLEK2	264.09	0
XBP1	134.70	0	P4HA1	48.91	2	CDC2	279.79	2
KRT18	136.35	0	BST2	50.56	2	VCAN	285.59	0
EEF1A2	138.25	0	F2R	52.22	1	FERMT1	286.92	1
SLC16A6	140.73	1	SPARC	52.86	1	MCOLN3	309.32	0
ACADSB	142.55	1	LDB2	56.29	2	TNFRSF21	315.68	1
SRD5A1	159.99	1	GJA1	58.54	0	KYNU	324.78	0
CHAD	164.19	0	PLAG1	60.29	1	TACC3	333.27	0
P4HTM	165.08	1	DSG2	68.03	1	TMC5	336.72	0

*Gene symbol not available, using accession number instead.

**Table 3:** Properties of six microarray meta-analysis methods.

	Rank average	mDEDS	Rank products	Choi	Wang	Naive (control)
Basic FS methods considered	FC, *T*, SAM, RS, mRMRQ/D	FC, *T*, SAM, RS, mRMRQ/D	FC^1^	*T* ^2^	FC^3^	FC, *T*, SAM, RS, mRMRQ/D
Chooses data-specific basic FS method(s)	*Yes*	No	No	No	No	*Yes*
Rank-Based	*Yes*	No	*Yes*	No	No	No

^1^Fold change between all interclass pairs of samples. ^2^Most similar to a *t*-statistic, but includes an estimate of interstudy variation. ^3^Computes a variance-weighted average of fold change. FC: Fold Change, *T* = *t*-statistic (*t*-test), SAM: significance analysis of microarrays, RS: rank sum test, mRMRQ/D: minimum redundancy, maximum relevance with quotient/difference.
