# Supplementary material for: Robust Microarray Meta-Analysis Identifies Differentially Expressed Genes for Clinical Prediction
Source: ScientificWorldJournal. 2012 Dec 18;2012:989637. doi: 10.1100/2012/989637 (PMC3539384; doi:10.1100/2012/989637)
Supplement: Supplementary file 1 — Supplemental Table S1 – Rating meta-analysis methods by prediction performance when combining all available datasets. This table lists the predictive performance (AUC, area under the ROC curve x 100) for each clinical application (breast cancer, renal cancer, and pancreatic cancer), data platform heterogeneity (Hom: Homogeneous, Het: Heterogeneous), and classifier (LR: Logistic Regression, DLDA: Diagonal LDA, and Linear SVM). The numbers in parentheses indicate the performance rating relative to other meta-analysis methods (rated horizontally, higher is better). A mean rating is computed for each clinical application and each meta-analysis method across all combinations of data platform heterogeneity and classifier. An overall mean rating is computed for each meta-analysis method. Ratings are proportional to bar lengths in Figure 3. [file 989637.f1.docx]

## Supplemental Table S1 – Rating meta-analysis methods by prediction performance when combining all available datasets

| **Combining Four Breast Cancer Datasets** | | | | | | |
| --- | --- | --- | --- | --- | --- | --- |
|  | **Rank Average** | **mDEDS** | **Rank Products** | **Choi** | **Wang** | **Naive** |
| **LR Hom** | 93.0 (4) | 91.1 (2) | 88.7 (1) | 93.0 (5) | 91.7 (3) | 93.6 (6) |
| **DLDA Hom** | 93.0 (4) | 91.9 (2) | 89.5 (1) | 93.1 (6) | 92.8 (3) | 93.1 (5) |
| **SVM Hom** | 91.5 (6) | 89.7 (2) | 87.5 (1) | 91.2 (4) | 90.2 (3) | 91.3 (5) |
| **LR Het** | 93.4 (5) | 91.7 (2) | 90.4 (1) | 93.7 (6) | 93.1 (3) | 93.2 (4) |
| **DLDA Het** | 93.1 (4) | 91.8 (3) | 91.6 (2) | 93.2 (6) | 93.2 (5) | 90.5 (1) |
| **SVM Het** | 92.3 (5) | 90.5 (1) | 90.5 (2) | 92.4 (6) | 91.9 (4) | 91.1 (3) |
| **Mean Rating** | **4.67** | **2** | **1.33** | **5.5** | **3.5** | **4** |
|  |  |  |  |  |  |  |
| **Combining Three Renal Cancer Datasets** | | | | | | |
|  | **Rank Average** | **mDEDS** | **Rank Products** | **Choi** | **Wang** | **Naive** |
| **LR Hom** | 97.5 (6) | 97.2 (3) | 95.1 (1) | 97.4 (4) | 96.8 (2) | 97.4 (5) |
| **DLDA Hom** | 97.0 (3) | 97.3 (4) | 95.7 (1) | 96.5 (2) | 97.4 (6) | 97.3 (5) |
| **SVM Hom** | 96.8 (5) | 96.4 (4) | 92.7 (1) | 96.9 (6) | 95.8 (2) | 96.2 (3) |
| **LR Het** | 96.8 (6) | 96.7 (5) | 95.3 (1) | 96.5 (4) | 96.5 (3) | 95.5 (2) |
| **DLDA Het** | 96.2 (4) | 97.0 (6) | 95.9 (2) | 96.0 (3) | 97.0 (5) | 95.6 (1) |
| **SVM Het** | 95.3 (5) | 95.2 (4) | 92.1 (1) | 95.6 (6) | 94.4 (3) | 94.2 (2) |
| **Mean Rating** | **4.83** | **4.33** | **1.17** | **4.17** | **3.5** | **3** |
|  |  |  |  |  |  |  |
| **Combining Three Pancreatic Cancer Datasets** | | | | | | |
|  | **Rank Average** | **mDEDS** | **Rank Products** | **Choi** | **Wang** | **Naive** |
| **LR Hom** | 81.5 (5) | 82.5 (6) | 80.6 (2) | 80.8 (3) | 80.9 (4) | 79.9 (1) |
| **DLDA Hom** | 81.4 (3) | 82.2 (5) | 82.4 (6) | 77.8 (1) | 78.2 (2) | 82.0 (4) |
| **SVM Hom** | 80.1 (6) | 79.9 (5) | 75.2 (1) | 79.5 (4) | 78.7 (2) | 78.9 (3) |
| **LR Het** | 78.8 (3) | 80.5 (5) | 79.7 (4) | 72.7 (2) | 81.3 (6) | 71.4 (1) |
| **DLDA Het** | 78.3 (3) | 80.2 (6) | 78.8 (4) | 69.7 (1) | 79.5 (5) | 70.5 (2) |
| **SVM Het** | 77.1 (5) | 77.3 (6) | 72.7 (3) | 71.3 (2) | 76.6 (4) | 68.8 (1) |
| **Mean Rating** | **4.17** | **5.5** | **3.33** | **2.17** | **3.83** | **2** |
|  |  |  |  |  |  |  |
|  | **Rank Average** | **mDEDS** | **Rank Products** | **Choi** | **Wang** | **Naive** |
| **Overall Mean Rating** | **4.56** | **3.94** | **1.94** | **3.94** | **3.61** | **3** |
| Performance is measured as area under the ROC curve (AUC) x 100. Numbers in parentheses indicate performance rating relative to other meta-analysis methods (higher is better). LR = Logistic Regression, DLDA = Diagonal LDA, SVM = Support Vector Machine, Hom = Homogeneous, Het = Heterogeneous | | | | | | |
